# Vessel segmentation for automatic registration of untracked laparoscopic ultrasound to CT of the liver

**DOI:** 10.1007/s11548-021-02400-6

**Published:** 2021-05-27

**Authors:** Nina Montaña-Brown, João Ramalhinho, Moustafa Allam, Brian Davidson, Yipeng Hu, Matthew J. Clarkson

**Affiliations:** 1 Wellcome/EPSRC Centre for Interventional and Surgical Sciences, University College London, London, UK; 2 Centre For Medical Image Computing, University College London, London, UK; 3 Division of Surgery and Interventional Science, University College London, London, UK

**Keywords:** Laparoscopic ultrasound, Vessel segmentation, Deep learning, Multi-modal registration

## Abstract

**Purpose:**

Registration of Laparoscopic Ultrasound (LUS) to a pre-operative scan such as Computed Tomography (CT) using blood vessel information has been proposed as a method to enable image-guidance for laparoscopic liver resection. Currently, there are solutions for this problem that can potentially enable clinical translation by bypassing the need for a manual initialisation and tracking information. However, no reliable framework for the segmentation of vessels in 2D untracked LUS images has been presented.

**Methods:**

We propose the use of 2D UNet for the segmentation of liver vessels in 2D LUS images. We integrate these results in a previously developed registration method, and show the feasibility of a fully automatic initialisation to the LUS to CT registration problem without a tracking device.

**Results:**

We validate our segmentation using LUS data from 6 patients. We test multiple models by placing patient datasets into different combinations of training, testing and hold-out, and obtain mean Dice scores ranging from 0.543 to 0.706. Using these segmentations, we obtain registration accuracies between 6.3 and 16.6 mm in 50% of cases.

**Conclusions:**

We demonstrate the first instance of deep learning (DL) for the segmentation of liver vessels in LUS. Our results show the feasibility of UNet in detecting multiple vessel instances in 2D LUS images, and potentially automating a LUS to CT registration pipeline.

## Introduction

Liver resection is the standard treatment with curative intent for patients with liver tumours, and is increasingly performed laparoscopically [1]. This approach has advantages over open surgery as less trauma is induced to the patient, resulting in shorter recovery times and reduced costs to the healthcare system [2]. However, it is estimated that only 5–30% of liver resections are performed laparoscopically—since the freedom of movement of laparoscopic tools is limited and there is no haptic feedback, patients with tumours that are large or close to major blood vessels are considered high risk for this procedure [1]. Laparoscopic ultrasound (LUS) is an imaging modality that can be used to visualise sub-surface structures which include tumours and vessels and thus increase the safety of laparoscopic surgery [3]. Given that LUS probes consist of a long shaft with a very small transducer at the tip, a high level of expertise is required from surgeons to both handle the probe and interpret their resulting images [4]. Additionally, some tumours are iso-echoic and thus not visible in LUS [5]. Therefore, registration of the LUS images to a pre-operative scan such as Computed Tomography (CT) using vessel information has been proposed as a guidance method—by combining both modalities, the surgeon can be provided with the spatial relationship between vessels and a target tumour.

The LUS to CT registration problem is particularly challenging and poorly constrained as the field of view of LUS is substantially smaller than that of CT. To mitigate this issue, the majority of proposed methods rely on an accurate manual initialisation to the registration along with electromagnetic (EM) tracking information to compose a LUS volume [6, 7]. However, the manual identification of anatomical landmarks for an initialisation disrupts the clinical workflow, and the introduction of tracking devices in the operating room is costly and logistically challenging. To enable clinical translation, we have previously proposed an untracked registration method based on Content-based Image Retrieval (CBIR) that replaces the need for a manual initialisation [8, 9], and presented results using manually segmented vessels. In this paper, we perform the first automatic vessel segmentation of 2D untracked LUS images using deep learning (DL), and show its potential in the automation of our registration pipeline.

### Background

Several authors have approached the vessel segmentation problem in ultrasound (US) across a range of applications. Vessel segmentation of 3D US has been performed by thresholding and filtering of Power Doppler images [10] as well as using adaptive thresholding with Hessian post-processing to extract vasculature from 3D B-Mode volumes [11]. However, there are no commercially available 3D LUS probes.

Other authors have proposed segmentation methods for 2D US, mainly for liver and musculoskeletal US. For musculoskeletal imaging, approaches have mainly focused on using temporal information across multiple images in a Kalman filter framework, while under the assumption that the imaged vessels have an approximately elliptical outline [12, 13]. However, this assumption is not compatible with the fact that the liver vasculature is complex and shows a highly variable appearance in 2D. For liver imaging, Song *et al.* [6] have segmented LUS images by using a Hessian based filter combined with the “Dip Image” mask proposed in [14], and filtering false positives by eliminating non-elliptical shapes. However, from our experience and data, many vessels in LUS do not show an elliptical outline, possibly limiting the applicability of this approach.

With recent advances in parallel computing, large-scale labelled datasets and advances in deep neural network architectures, deep learning based approaches using convolutional neural networks (CNNs) have been applied to 2D US hepatic vasculature segmentation with growing success. A patch-based CNN method with post-processing using k-means clustering was proposed to detect vessels in abdominal 2D US [15], but UNet [16] methods have proven to achieve higher segmentation performance with small, abdominal 2D US datasets [17] and abdominal 3D US datasets [18]. To date, there are no applications of DL to LUS segmentation present in the literature. Given the limited availability of clinical LUS liver data, we propose the use of UNet for the LUS vessel segmentation task, and assess its feasibility as part of an automatic untracked LUS to CT registration framework.

### Contributions

In this paper we report the first instance of 2D LUS liver vessel segmentation using a UNet and integrate it in a registration pipeline. Our contributions include:

- The first LUS vessel segmentation results on clinical data using DL.
- An evaluation of performance bounds for UNet for LUS vessel segmentation on clinical data.
- A novel measure to evaluate image-wise multiple vessel detection.
- The first results of a fully automatic initialisation to the untracked LUS to CT registration problem.

## Methods

### Data description

LUS images with 668 $\times$ 544 pixels and pixel size 0.12 $\times$ 0.12 mm from 6 patients were acquired intra-operatively at a frequency of 40 Hz by smoothly sweeping a BK Medical 4 Way I12C4f probe^1^ over the surface of the liver. A slow moving LUS probe, sampling at 40 Hz results in very similar sequential frames. We obtain sets of distinct images for each patient by selecting every fifth frame as neighbouring frames acquired at high frequency are highly correlated. To obtain ground truth vessel labels, vessels were manually segmented in each image. The resulting labelled sample size is described in Table 1.

**Table 1:** Number of vessel labelled LUS images per patient

Patient number	1	2	3	4	5	6	Total
Total number of images	173	248	46	134	704	539	1844

### LUS vessel segmentation model

A modified 2D UNet [16] with batch-norm blocks [19] at each layer was trained using a differentiable Dice loss to segment vessels from LUS images. To avoid leakage of information between datasets, the dataset was separated by patient, and models were trained with combinations of (4, 1, 1) patients in train, validation, and testing sets. The data was augmented using 2D transformations with a random rotation between $[-10, 10]^\circ$, a crop between sizes [0.9, 1.0], and a random left-right flip. To reduce the patient-wise dataset size imbalance, the individual patient datasets were upsampled before data augmentation to match the number of images in the largest patient set in the training set. The upsampling was performed by random repetition of images in the training set.

### Segmentation performance evaluation

In addition to the model, we describe the measurements used to quantify vessel segmentation performance.

Dice score is commonly used as a global performance metric for segmentation methods [20] and has been used to quantify performance in other hepatic US vessels segmentation methods [17, 18]. For a ground truth segmentation label *L* and associated prediction *P*, we measure the binary Dice score *D*:

1 $$\begin{aligned} D(P, L) = \frac{2 |L \cap P|}{|P| + |L|} \end{aligned}$$

In the proposed LUS to CT registration framework, like most existing algorithms in this application, topological features of the extracted vessels, such as the number, size, shape and locations, are of importance for aligning corresponding anatomical locations. Therefore, the segmentation performance analysis should also consider if these topological features are accurately detected locally.

We propose to assess the individual vessel detection rate per image by comparing prediction and ground truth vessels with a criterion based on a closest distance matching and a vessel size ratio. Given a prediction *P*, we define the success $S_{l}$ in the detection of a ground truth vessel in *L* with centroid $c_l$, calculated using the image moments of the contour *L*, through the following condition:

2 $$\begin{aligned} S_{l} = {\left\{ \begin{array}{ll} 1, &{} \text {if } ||c_{l} - m(c_{l}, P)||^2 < 2r_{l} \\ &{}\text { and } {\text {min}}\big (\frac{r_l}{r_p}, \frac{r_p}{r_l}) > 0.5 \\ 0, &{} \text {otherwise.} \end{array}\right. } \end{aligned}$$

In this equation, $m(c_l,P)$ defines a closest match operator that returns the position of the predicted vessel that is closest to $c_{l}$ in terms of Euclidean distance, and $r_l$ represents the equivalent radius of a vessel with centroid $c_l$ and area $A_l$, such that $r_l = (\frac{A_l}{\pi })^{\frac{1}{2}}$. We consider a ground truth vessel with equivalent radius $r_l$ to be successfully detected if the prediction detects a vessel with a centroid within a distance below 2$r_l$, and where either $r_p$ or $r_l$ are more than half the size of the larger radius counterpart. This constrains the radius search such that the vessels must be within a certain size tolerance to be determined as correctly segmented.

Considering that each image has *N* ground truth vessels, we define the detection rate as the percentage of vessels that are successfully detected in *P*:

3 $$\begin{aligned} S_{P} = \frac{\sum _{l=1}^{N}{S_{l}}}{N} \end{aligned}$$

### Integration of segmentation in a registration framework

To further validate our segmentation, we integrate our results in our previously developed untracked LUS to CT registration framework [8, 9]. In summary, this method comprises two main steps, an image retrieval step and a probabilistic optimisation. For image retrieval, we first generate a possible set of registration solutions by intersecting the segmented CT vessel models with multiple LUS planes parameterised by virtual probe poses, and encoding the resulting 2D vessel maps into feature vectors consisting of vessel position and area. The resulting feature vector-pose pairs are stored in a database and grouped according to number of vessel occurrences. Given a segmented LUS image, registration is then achieved by finding the pose that shows the best content match in the database.

Since the vessel matching problem between LUS and CT is ambiguous, we employ a probabilistic optimisation to combine the retrieval results of multiple LUS images in time. Considering that images that are close in time should also be close in rotation and translation, we use a discrete Hidden Markov Model (HMM) to estimate the most likely sequence of database poses to represent the LUS acquisition in CT space. We refer the reader to [9] for further details.

## Experiments

We perform three sets of experiments to evaluate the performance of our framework. All of our UNet models were trained on NVIDIA Quadro P5000 GPUs for 1000 epochs, using an Adam optimiser with a learning rate 10$^{-5}$, and a batch size of 32. Models were evaluated at convergence according to lowest validation loss for each model during training.

In a first experiment, we trained separate models for the segmentation of each of the six patient datasets and assess their performance using the Dice score as in Sect. 2.3. Given the limited amount of data in our sample across the 6 patients (see Table 1), we expect the LUS image dataset from each patient to show distinct vessel size distributions. Therefore, to assess the impact of dataset size and vessel shape distribution on segmentation model performance, we perform a sixfold nested cross-validation (CV) with 5 inner folds by holding out a different patient for each outer fold as described in Sect. 2.2. An analysis of variance (ANOVA) is performed to test whether each of the 5 inner fold models per hold-out test patient are significantly different from each other in terms of mean Dice score. This means that for each patient, we test whether excluding a different patient dataset from the training influences the final model. In the second experiment, we measure the detection rate (as defined in Sect. 2.3) of the best mean Dice score performing models per patient, and assess the effect of vessel size on the detection performance.

In a third and final experiment, we perform untracked registrations using segmentations from the best mean Dice scoring models. To mitigate the effect of segmentation noise, we remove segmentations that amount to less than 120 pixels. Liver surface and vessel models are extracted from contrast enhanced CT scans using a commercial service.^2^ We do not consider patients 2 and 4 in this experiment. In these cases, we observed that the CT segmented models did not show certain vessels that were clearly visible in LUS—which can occur due to the timing of the CT contrast enhancement—impeding an accurate match for registration. For each patient, we register 5 sweeps of 6 LUS images using the single label registration CBIR formulation in [9] and 200 retrieved poses. Compared to this work, we keep all parameters the same, apart from two range intervals in the CBIR simulation step: we constrain the probe rotation across the liver surface to be in the range $[-90,0]^\circ$, and the probe placement across the liver surface normal to be in the range [10, 25] mm. To evaluate registration performance, we define a ground truth registration by manually picking vessel landmarks in the LUS planes, their counterparts in CT and performing a point-based alignment, as described in [9]. Registration accuracy is then assessed as the Target Registration Error (TRE) between the position of LUS landmarks predicted by the output registration and the ones of CT.

## Results

### Dice score and model variability analysis

**Fig. 1:**
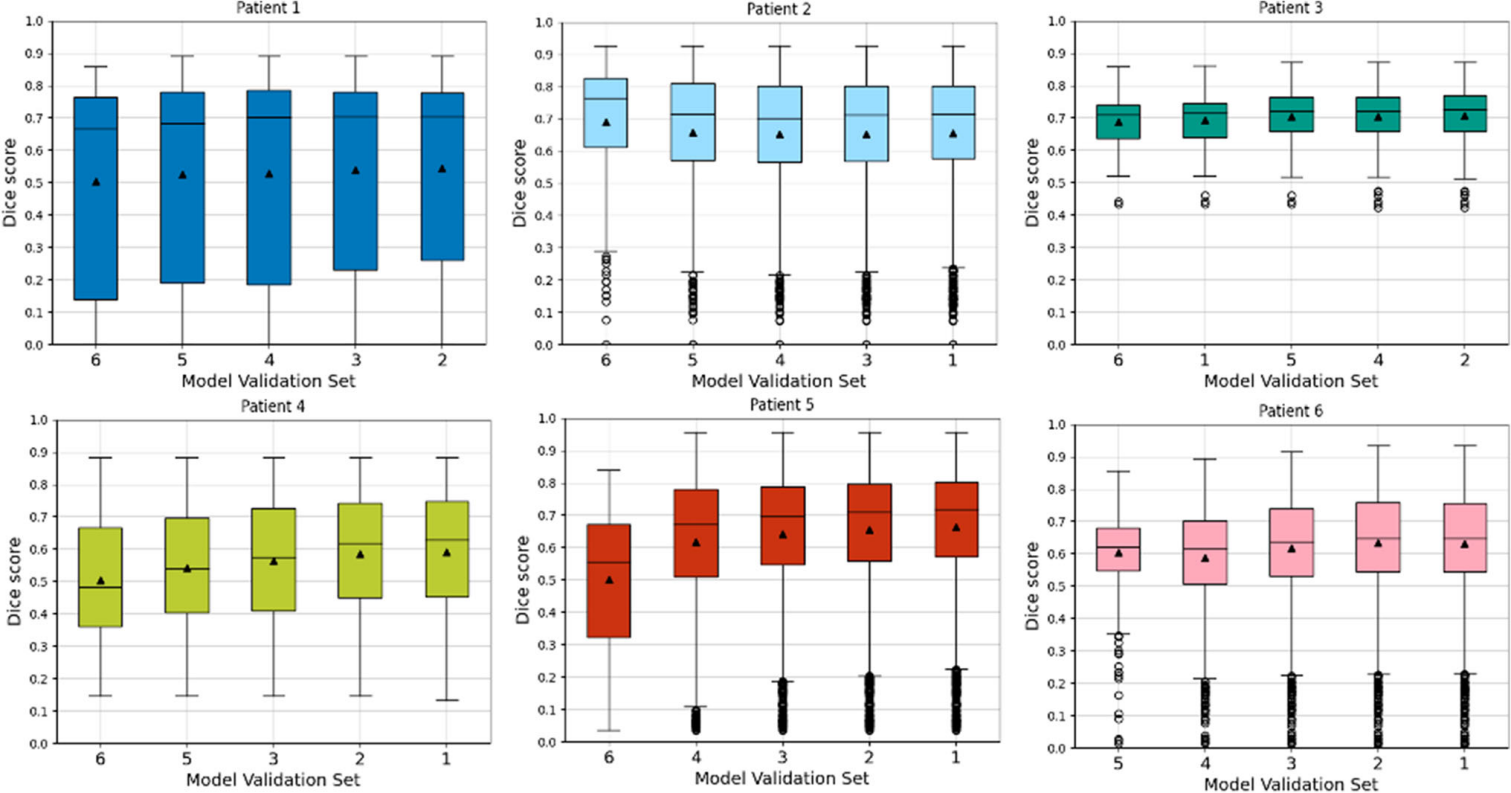
Inner fold CV Dice score distributions obtained for each of the 6 outer folds of the nested CV. Black line and black triangle refer to median and mean Dice score, respectively

**Table 2:** Best mean Dice score per hold-out test patient, and respective validation set

Test patient	1	2	3	4	5	6
Validation patient	2	6	2	1	1	2
Mean Dice score	0.543	0.689	0.706	0.592	0.665	0.634

**Table 3:** Models with significantly different mean Dice scores by validation and test sets

Val(A)	Val(B)					
	1	2	3	4	5	6
1	X	–	–	–	4, 6	4, 5
2	–	X	–	–	4, 6	4, 5
3	–	–	X	5, 6	–	4,5
4	–	–	–	X	–	5
5	–	–	–	–	X	–
6	–	–	–	–	–	X

In each row and column, the possible validation patient sets used in the pairwise tests are displayed as Val(A) and Val(B). Each cell shows the outer fold test patients for which the pairwise t-test resulted in a *p*-value below the Bonferroni corrected $\alpha = 0.0008$, denoting statistical significance

We report two sets of results for our first experiment. Firstly, we present Dice score distributions from each of the 6 outer fold test set as boxplots in Fig. 1. In each chart, we present the results for each of the 5 inner folds, separated by the patient used as the validation set during training in the CV. Additionally, we report the highest mean Dice score based on the inner fold model for each of the outer fold test patients in Table 2. Highest Dice score values are obtained for test Patient 3, where a maximum mean of 0.706 is observed. Test patients 2, 5, and 6 show lower mean Dice score results, with maximums ranging from 0.634 to 0.689. A poorer performance is observed for test patients 1 and 4. In terms of variance, models from Patient 1 show the highest Dice score variance and the ones from Patient 3 the lowest.

In order to assess the model variability, we report the combinations of significantly different Dice score performing models for each outer fold in Table 3. In this table, model combinations represented by validation sets A (row), B (column) and outer fold (cell) that achieve a *p*-value under the Bonferroni corrected $\alpha =0.0008$ from a pairwise t-test are reported. Statistically different models are observed for test patients 4, 5 and 6. Additionally, these differences also arise mainly when the compared models do not contain these patients in their training subsets.

### Vessel detection rate

**Fig. 2:**
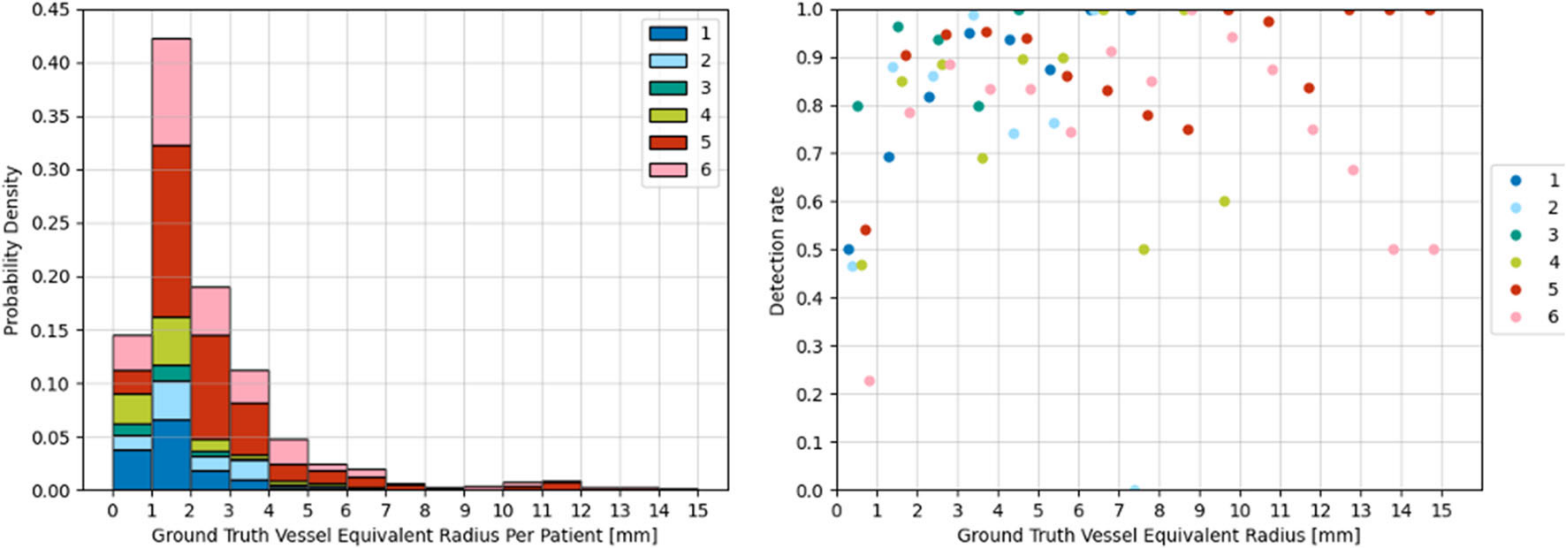
Detection rate per vessel equivalent radius for the best performing Dice models per patient. Left displays histograms with the number of vessel occurrences per equivalent radius for each patient dataset are presented. Right displays the corresponding detection rate per equivalent radius. Equivalent radii values are binned in 1 mm intervals

Vessel detection rates for the best performing Dice score models are reported in Table 4. Compared to the best Dice values of Table 2, there is a clearer separation in performance across patients—Patient 3 is the highest performing with a mean detection rate of 0.885, consistent with Dice score performance, whereas Patient 4 shows a lowest mean rate of 0.412.

**Table 4:** Mean and standard deviation of vessel detection rate obtained by best Dice performing models per patient

Test Patient	1	2	3	4	5	6
Detection rate (mean)	0.681	0.818	0.885	0.412	0.877	0.610
Detection rate (std)	0.248	0.220	0.099	0.177	0.155	0.225

**Fig. 3:**
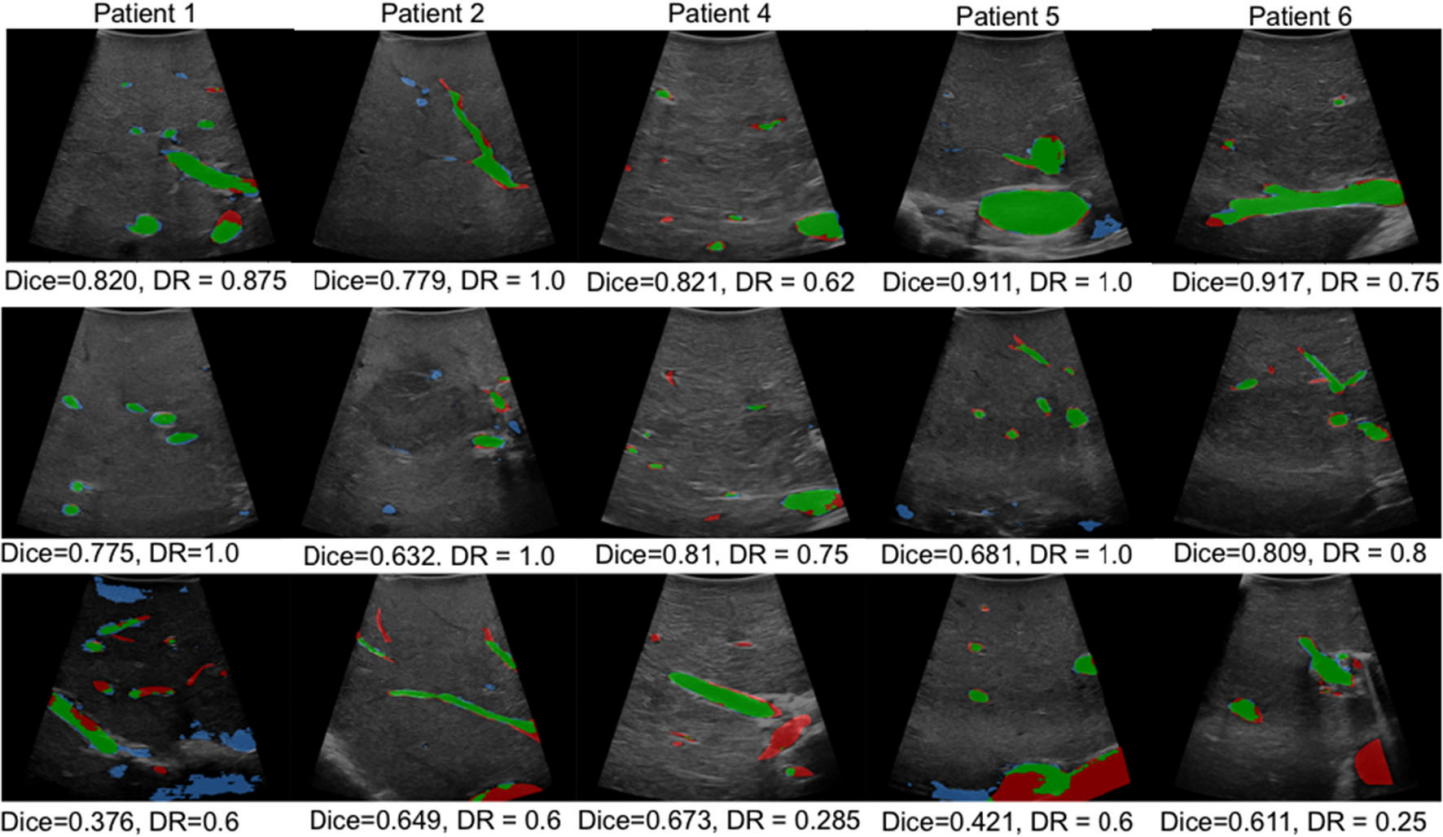
Segmentation results from UNet models for different hold-out patients. True positive vessel segmentations are overlaid in green, false positives in blue and false negatives in red. DR stands for image detection rate

Results of the effect of ground truth vessel size in the detection rate are shown in Fig. 2. In this case, instead of measuring the detection rate per image $S_{P}$ (see Eq. 3), we measure the detection rate individually for all vessels with an equivalent radius within fixed intervals of 1 mm across the whole sample. Therefore, in the left plot we show a histogram with the distribution of vessel sizes represented by the equivalent radius for each patient set, and in the right we show the respective vessel detection rates for each bin. Highest detection rates of 70 to 100% are observed across all patients for vessels with radius between 1 and 6 mm, and a peak maximum is observed in the 2–3 mm bin, which coincides with the statistical majority of vessel feature sizes as in Fig. 2. For radii above 6 mm, the detection rate shows a decrease in some patients and becomes overall more variable. Visual results of some of these segmentations are displayed in Fig. 3 with their respective Dice score and detection rate. In each image, false negative segmentations are marked with red, false positive with blue, and true positive with green. In the top row, we show examples that result in a high Dice score, whereas in the middle we show examples with a high detection rate. These examples show that Dice score has a higher value when segmenting larger vessel sections, whereas detection rate is more influenced by the number of detected vessels. In the bottom row we show examples of poor segmentations, mainly in images with larger vessel sections.

### Registration

**Fig. 4:**
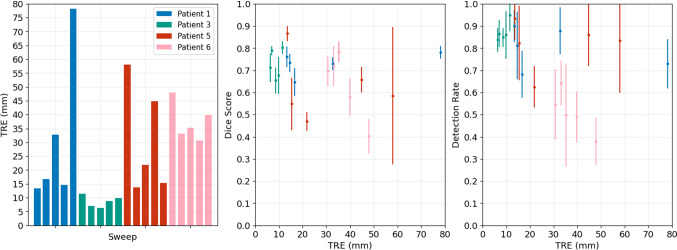
Registration accuracy results of 5 LUS sweeps from 4 patients. Left chart shows TRE. Middle and right charts show the mean and standard deviation of Dice score and detection rate of the registered LUS segmentations compared to sweep TRE, respectively

Registration accuracy measurements of 5 sweeps of 6 LUS images for 4 patients are presented in Fig. 4. In addition to TRE shown in the left chart, we also show the Dice score and detection rates per sweep against TRE in the middle and right charts, respectively. Considering that our registration algorithm follows a global optimisation and should be used mainly as a registration initialisation tool, we consider a TRE below 20 mm to be of clinical value. Following this criterion, best results are obtained for Patient 2, where the TRE ranges from 6.3 to 11.4 mm. In the cases of Patients 1 and 5, acceptable TRE values ranging from 13.3 to 16.6 mm are obtained in 3 and 2 sweeps out of 5, respectively. No acceptable TRE values are obtained in the sweeps of Patient 6. Detection rate measurements show a reasonable agreement with the TRE—all but one of the sweeps registered with a TRE below 20 mm show a mean detection rate above 0.80, and lower mean rates between 0.37 and 0.64 result in poor registrations. Dice score values are less distinct across registration accuracy, showing values between 0.54 and 0.86 for the accurate cases.

**Fig. 5:**
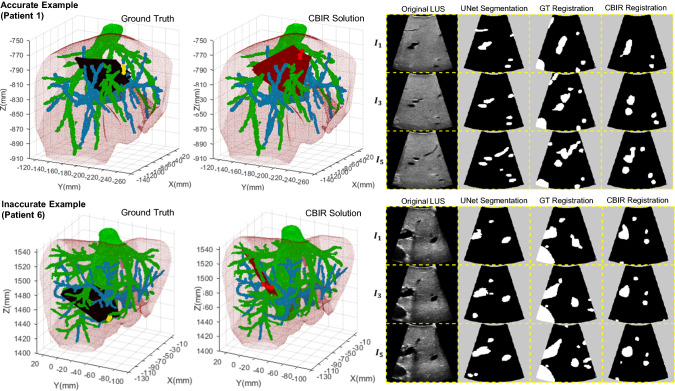
Visual results from two registration examples. Left shows the 3D visualisation of the ground truth (black planes) and the CBIR registration solution (red planes), whereas right shows the corresponding 2D slicing results of 3 images in the sweep. The column “GT registration” refers to the ground truth manually aligned LUS plane in CT, and “CBIR Registration” refers to the CT database plane that matches the UNet segmentation most closely according to the CBIR algorithm. The accurate example has a Dice (DR) score of 76 (90)% with TRE of 13.31 mm, and the inaccurate example has a Dice (DR) score of 58 (49) % with TRE of 39.82 mm

Visual results of two registration examples are displayed in Fig. 5: in the top, we show an accurate registration with a TRE of 13.3 mm for Patient 1, and in the bottom we show an inaccurate registration with a TRE of 39.8 mm for Patient 6. The 3D result of our solution and the ground truth is presented in the left, and the corresponding 2D vessel projections are shown in the right for 3 images in the registered sweep. In the case of Patient 1, an accurate segmentation leads to a solution that is topologically consistent with the ground truth. However, in the case of Patient 6, the failure in segmenting a large vessel section leads to a topologically inaccurate registration.

## Discussion

Our results show that UNet is suitable for liver vessel segmentation in LUS images. Our Dice score performances, the first reported for LUS segmentation, are comparable to other DL-based US vessel segmentation methods in the literature [17, 18]. Even though there are other models in the literature, UNet is still the most appropriate approach to deal with limited size medical image datasets. Additionally, recent work has demonstrated that a general automatic hyperparameter tuning of a standard UNet architecture can improve results further for the majority of datasets [21]. Further registration experiments also highlight the potential of UNet in automating a registration pipeline—in 50% of cases, clinically valuable accuracies ranging from 6.2 to 16.6 mm were obtained.

The ANOVA reveals that models trained with larger patient datasets achieve higher Dice scores with lower variances. Specifically, models trained without the larger datasets from patients 4, 5, and 6 which hold 134, 704 and 539 images respectively, showing differing performance with statistical significance. An exception was observed for Patient 1 (see Fig. 1), where a larger intra-model variance in segmentation results is observed. This can be explained by the fact that almost half of the images in this sample were acquired with different contrast settings. This is highlighted in the visual result of Fig. 3, where the bottom row figure of Patient 1 has a poor performance.

The ground truth distribution of vessel sizes could explain the varying performances for larger vessels in Fig. 2—models are better at detecting instances similar to the majority feature in the training set. Whilst smaller vessels are more consistently detected by our models, Dice scores are lower for segmentations of smaller areas despite high detection rates (see Fig. 3). Overall, this experiment suggests that UNet generisability can be increased by supplying models with more labelled data with a wide feature distribution across vessel sizes, a well-known trend in DL methods.

Our registration results are in agreement with previous trends in Dice score and detection rate—the best performing segmentations of Patient 3 result in the best registration accuracies with values below 10 mm, and the worst performing segmentations of Patient 6 do not result in any clinically usable registration. These results also suggest that our detection rate measure is more informative than Dice score for registration performance. In almost all of the clinically usable registrations, detection rate showed high values, whereas Dice shows less distinct values across different registration accuracies. A few exceptions are observed mainly for Patient 5, where high detection rates have resulted in poor registrations. This may be explained by the poor segmentation of larger size vessels (see Fig. 2)—even if the majority of vessels are detected, the failed detection of a major vessel can lead to an inaccurate registration, as shown in the inaccurate example of Fig. 5.

For future work, we propose three directions to improve segmentation performance. Firstly, we intend to include a larger cohort of patient data with a wider distribution of vessel sizes across each patient. Secondly, given the performance variability across different vessel sizes, we aim to refine our loss to specifically penalise mis-segmentations in larger vessels that are likely to be more relevant for registration. Lastly, we aim to incorporate time-series sequential constraints on the 2D segmentation problem. By using a recurrent model such as Mask-RCNN [22], the segmentation of rapidly varying vessel sections over time could be improved.

## Conclusion

We present the first DL framework for the segmentation of vessels in untracked 2D LUS liver images, and integrate them in a untracked LUS to CT registration pipeline. Despite the size of our sample, our segmentation shows mean Dice scores ranging from 0.543 to 0.706, results that are comparable with current state-of-the-art approaches. Additionally, we observed that performance improves by considering larger training datasets with a wider distribution of 2D vessel shapes. Using these segmentations, we are able to obtain untracked registrations with accuracies ranging between 6.3-16.6 mm. Therefore, we also demonstrate for the first time fully automatic pipeline for the coarse registration of LUS to CT, potentially enabling the clinical translation of this image-guidance technique in the future.

## Footnotes

1 www.bkmedical.com.

2 https://www.visiblepatient.com.
